# A Rare Case of Loperamide-Induced Cardiac Arrest

**DOI:** 10.7759/cureus.9396

**Published:** 2020-07-26

**Authors:** Sowjanya Kapaganti, Shahryar Anwar Ansari, Richard Saba, Ahmed Elkhouly, Mohab Hassib

**Affiliations:** 1 Internal Medicine, St. Francis Medical Center, Trenton, USA

**Keywords:** ventricular arrhythmia, qtc prolongation, arrhythmias, loperamide, opioid abuse

## Abstract

Loperamide (Imodium) is an opioid receptor agonist available over-the-counter and can be used for the treatment of diarrhea. When ingested in excessive doses, loperamide can penetrate the blood-brain barrier and is reported to produce euphoria, depression of the central nervous system, and cardiotoxicity. It may also be used for its euphoric effects and potentially to alleviate opioid withdrawal. Loperamide has a US boxed warning for torsades de pointes and sudden death. Loperamide has been reported to cause torsades de pointes, cardiac arrest, and death when higher than the recommended dosage is consumed. We report a rare case of ventricular arrhythmia provoked by accidental ingestion of loperamide to treat simple diarrhea.

## Introduction

Few medications may be perceived as relatively harmless because they are over the counter, however, all medications need to be taken cautiously and as directed to avoid major adverse effects. Consuming a higher than the recommended dose of a simple antidiarrheal, such as loperamide, can lead to prolongation of the corrected QT interval (QTc). This can manifest as torsades de pointes, ventricular fibrillation (VF), and sudden cardiac death. This risk is augmented in the presence of electrolyte imbalances that further prolong the QTc and increase the risk for sudden cardiac death. Loperamide has a US boxed warning for such conditions[1-3].

## Case presentation

A 38-year-old female patient without any relevant past medical history or family history was brought to the emergency department by the emergency medical service (EMS); she was found to be unresponsive, and the downtime was estimated around 30 minutes. The patient had been suffering from flu-like symptoms, diarrhea, and vomiting for one week before hospitalization. She took an unknown amount of loperamide for diarrhea. As per her husband, he found two empty bottles of loperamide that he suspected his wife has consumed over approximately 48 hours. 

**Figure 1:**
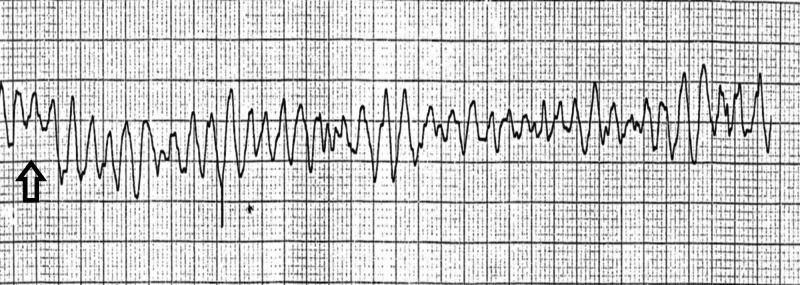
EMS telemetry strip at the time of the arrest showing ventricular fibrillation (arrow) EMS: emergency medical service

Since the patient was unresponsive, her husband started cardiopulmonary resuscitation (CPR) until the EMS arrived at the scene; the initial rhythm reported by the EMS was VF (Figure 1). Two shocks were delivered via defibrillation mode with continuous CPR as per advanced cardiac life support (ACLS) protocol following which return of spontaneous circulation (ROSC) was successfully achieved.

**Figure 2:**
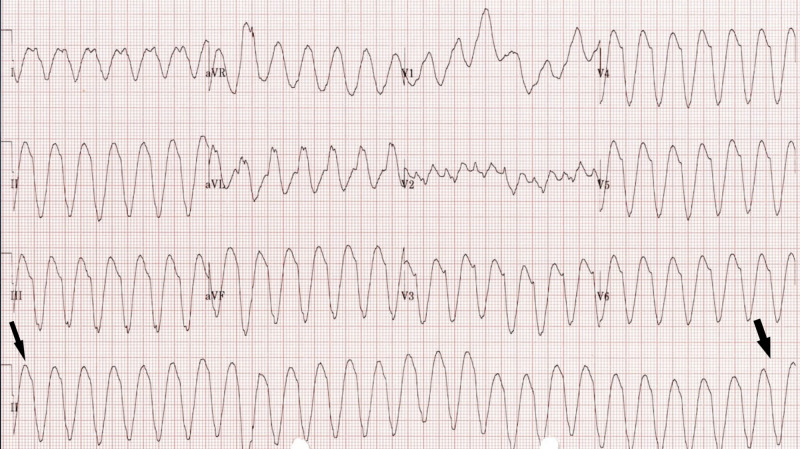
Electrocardiogram (EKG) revealing monomorphic ventricular tachycardia in the hospital (arrows - lead II on EKG)

The patient was admitted to ICU where admission workup showed a prolonged QTc of more than 600 mSec on her electrocardiogram (EKG) and elevated troponins (Figure 2). CT-angiography was performed showing no evidence of pulmonary embolism. Urine drug screen done did not demonstrate any illicit drug use. The patient was started on an amiodarone drip, despite which she had bouts of sustained ventricular tachycardia and another episode of VF for which she received a shock via defibrillator. Emergent cardiac catheterization revealed non-obstructive coronaries. Bedside echocardiography showed an ejection fraction of 45%-50%. She was then switched to a lidocaine drip, given her persisting episodes of ventricular tachycardia persisted despite being on amiodarone. A multidisciplinary team discussion with cardiology, critical care physician, an internist was held following which lidocaine was discontinued and VF was traced back to loperamide under the setting of electrolyte abnormalities. Meticulous electrolyte repletion to maintain potassium of 4 and magnesium of 2.5 was done as that could have potentially been aggravating factors in her prolonged QTc. 

With optimal electrolytes, the patient’s condition stabilized with no further episodes of ventricular tachycardia. A repeat EKG showed that QTc had shortened to less than 500 msec within 24 hours. For secondary prevention, the patient received an implantable cardioverter (ICD).

After extubation, the patient was found to have a hypoxic brain injury, a long term sequelae likely from the cardiac arrest-induced brain hypoxia. Over day 9 of the hospital course, her neurological status had started to gradually improve, and she was transferred to a neurology rehabilitation institution. 

## Discussion

In supratherapeutic doses, loperamide can cause life-threatening cardiac arrhythmias due to QRS and QT segment prolongation which can lead to ventricular tachycardia [1]. Loperamide is used for acute and chronic diarrhea and the maximum dosage should not exceed 16 milligrams per day. Dosages beyond this can lead to serious morbidity. In 2016, the Food and Drug Administration (FDA) issued a warning about potential severe side effects including cardiac dysrhythmias [2]. Loperamide exhibits similar action to Vaughan-Williams class IA, III, and IV antiarrhythmics, with dose-dependent effects on the hERG/Ikr potassium channel, cardiac sodium channels, and L-type calcium channels [1]. The most serious effect of blocking cardiac sodium channels is a prolongation of the QRS segment, polymorphic ventricular tachycardia, torsades de pointes, and sudden cardiac death [1]. In some case reports, loperamide-induced arrhythmia was found to be refractory to magnesium sulfate, amiodarone, sodium bicarbonate, potassium chloride, fatty acid emulsion, cardioversion, and repeated defibrillation [1]. In these refractory cases, overdrive electrical pacing or isoproterenol continuous infusion may be beneficial [1].

Even when used at the recommended therapeutic dosages, patients can experience ventricular tachycardia and syncope. Due to this, physicians should avoid loperamide in those with known baseline QT segment prolongation, for those with risk factors for QT prolongation, with other drugs that cause QT prolongation, or with electrolyte abnormalities. In our case, the patient had potentially ingested two bottles or more of loperamide with electrolyte disturbances likely due to diarrhea. Her ventricular tachycardia did not respond to medications alone, however, defibrillation did aid in terminating her VT. VT recurred again when she arrived at the hospital and at that stage, her electrolyte abnormalities were noted.

The half-life of loperamide is variable depending on the dosage taken. The usual half-life of loperamide is approximated to be between nine and thirteen hours, however, half-lives of greater than forty hours have been documented with doses of 16 milligrams [2]. This is likely due to several mechanisms. One mechanism is that at higher dosages loperamide can cross the blood-brain barrier [1] and lead to a greater bodily distribution of loperamide which prolongs the excretion of the drug. Another reason for its prolonged half-life is that loperamide is a mu-receptor agonist, which causes it to decrease gastrointestinal motility and slow the rate of absorption of the drug [3]. Loperamide also exhibits low oral bioavailability, and extensive first-pass hepatic metabolism [1]. This fact meant that the use of cytochrome P450 3A4 inhibitors, which is a characteristic of several commonly used drugs, can decrease the hepatic metabolism of loperamide and prolong its half-life [1]. Therefore, the metabolism and excretion of loperamide can be manipulated by several factors. In the setting of serious adverse events such as cardiac arrhythmia, slower excretion of loperamide is unfavorable, as the causal factor lingers within the body, potentially worsening the outcome.

Loperamide has become more recognized as a potential drug of abuse. With the opioid epidemic that is ongoing, opioids have been classified as controlled drugs. There exists more pressure on prescribers to limit the number of opioids given both in hospitals and in the community. Furthermore, physicians and pharmacists are increasingly becoming more educated on drug-seeking behaviors. These circumstances make it more difficult for those looking to abuse opioids. As mentioned above, loperamide can cross the blood-brain barrier at high doses leading to euphoria and central nervous system depression as it acts on the same receptors as opioids. Many opioid abusers are turning to this inexpensive over the counter medication as an alternative to opioids or to temporarily self-treat their withdrawal symptoms. The National Poison Data System have shown a national increase in loperamide misuse [4]. Although this national increase in misuse was deemed as intentional misuse, the case described above is unlikely to describe deliberate overdose of the drug, however, the adverse events are the same regardless of the intention behind the overdose. For this reason, patients should be warned about exceeding the maximum daily dose, especially in the community.

## Conclusions

We concluded that this patient’s VF and sudden cardiac arrest were due to the loperamide overdose under the setting of electrolyte imbalance. Loperamide and electrolyte imbalance can independently cause cardiac dysrhythmia, however, when paired together the risk for cardiac rhythm disturbance is much higher. We also wanted to emphasize educating the general population by the health care providers to be more mindful of the adverse effects of many simple over-the-counter medications such as loperamide which could be detrimental.
